# Supplementary material for: Galaxy-ML: An accessible, reproducible, and scalable machine learning toolkit for biomedicine
Source: PLoS Comput Biol. 2021 Jun 1;17(6):e1009014. doi: 10.1371/journal.pcbi.1009014 (PMC8213174; doi:10.1371/journal.pcbi.1009014)
Supplement: S1 Text — (DOCX) [file pcbi.1009014.s001.docx]

For this benchmark we trained and evaluated a variety of supervised machine learning pipelines using 164 classification datasets with both binary and multi-class classifications, and 112 datasets with real-values targets from the Penn Machine Learning Benchmark repository [1] (Link: <https://github.com/EpistasisLab/penn-ml-benchmarks>). S1 Table provides links to Galaxy workflows and histories created, and all analyses can be precisely reproduced. The Penn ML benchmark analysis in Galaxy has two parts—classification and regression.

For classification, two Galaxy histories are shared: (i) classification performance of 15 classifiers with default parameter values on 164 datasets and (ii) the same classification analysis but with optimised parameters. In this analysis, XGBoost classifier outperforms other classifiers (Fig 2) by obtaining the best performance on most of the 164 datasets. Links to the workflows for XGBoost classifier with the default and optimised parameters are listed in S1 Table. For example, the workflow for XGBoost classifier to achieve the best performance has two steps:

1. Preprocessing: scales all the datasets using scikit-learn’s RobustScaler.
2. SearchCV: optimises the hyperparameters such as the *number of estimators, learning rate,* and *max_depth* of XGBoost classifier.

For regression analysis, we created one Galaxy history to measure the performance of 14 regressors with the default and optimised parameters on 112 datasets. S1 Table lists this history and associated workflows. Similar to our classification results, the XGBoost regressor records the best performance (see S1 Fig). For example, the XGBoost regressor workflow achieving the best performance has one step (named SearchCV) that optimises the hyperparameters such as the *number of estimators, booster* and *max_depth* of XGBoost regressor. For each hyperparameter, a range of values is specified and using grid search, all parameter combinations are tried, and performance is reported with the optimal parameter settings. All the resulting datasets from running regression algorithms with and without parameter optimization is available from: <https://usegalaxy.eu/u/kumara/h/pmlbregressionanalysisjune2020>.

**References**

1. Olson RS, La Cava W, Orzechowski P, Urbanowicz RJ, Moore JH. PMLB: a large benchmark suite for machine learning evaluation and comparison. BioData Min. 2017;10: 36.
