# Supplementary material for: Galaxy-ML: An accessible, reproducible, and scalable machine learning toolkit for biomedicine
Source: PLoS Comput Biol. 2021 Jun 1;17(6):e1009014. doi: 10.1371/journal.pcbi.1009014 (PMC8213174; doi:10.1371/journal.pcbi.1009014)
Supplement: S2 Text — (DOCX) [file pcbi.1009014.s002.docx]

Using tools for data preprocessing, feature selection and regressors, we performed an aggregated analysis on 112 regression datasets from Penn Machine Learning Benchmark repository. This repository contains numerous datasets for regression many of which are of biological importance. We applied 14 different regressors on 112 datasets from the collection and performed a detailed comparison of performances of these regressors (S1 Fig). To measure the accuracy of regression models, we used the r-squared metric (R2), which is common in regression analyses. This metric can be any real number to a maximum of 1.0. If it is negative, it suggests that the regression model is not good. If it is closer to 1.0, the model’s performance is good. We used 5-fold cross-validation for training and repeated it for 10 experiment runs to compute a mean r-squared score for each dataset. We achieved a r-squared score of more than 0.80 for 3 regressors (xgboost, gradient boosting and extra trees) and close to 0.80 for 2 regressors (bagging and random forest).
