# Supplementary material for: Galaxy-ML: An accessible, reproducible, and scalable machine learning toolkit for biomedicine
Source: PLoS Comput Biol. 2021 Jun 1;17(6):e1009014. doi: 10.1371/journal.pcbi.1009014 (PMC8213174; doi:10.1371/journal.pcbi.1009014)
Supplement: S3 Text — (DOCX) [file pcbi.1009014.s003.docx]

In our second analysis, we analyzed cancer cell lines gene expression and drug response datasets from the Cancer Dependency Map Project [1] (<https://depmap.org/>). This dataset includes more than 50,000 gene expression values for over 1000 cancer cell lines obtained from bulk RNA-seq as well as drug response data for 265 drugs. Both gene expression data and drug response targets are continuous data. We had several goals in mind when performing this analysis. We wanted to assess how well supervised learning performed on a dataset with a very large number of features and a relatively small and imbalanced number of examples. These challenges are common when machine learning is applied to molecular datasets. We also wanted to compare performance of meta-ensemble (stacking) approaches with traditional single-model methods.

- Galaxy History URLs:
  - Regression: <https://usegalaxy.eu/u/qiang_gu/h/depmap-regression>
    - Example workflow: <https://usegalaxy.eu/u/kumara/w/stackingensembleregressorpcaknr>
  - Classification: <https://usegalaxy.eu/u/qiang_gu/h/depmap-classification>
    - Example workflow:<https://usegalaxy.eu/u/kumara/w/stackingclassifierdrugprna2>

Because target values for this analysis—cell line drug response data—was continuous, we developed a strategy to binarize the data so that classification approaches could be used. Drug response values were standardized/z-scored, and:

- cell lines with a standardized value of less than -1 were labeled responders;
- cell lines with a standardized value between -1 and 0 were labeled indeterminate;
- cell lines with a standardized value greater than 0 were labeled nonresponders.

We implemented this strategy in two tools in the Galaxy-ML utils library, binarize_average_precision_scorer and binarize_auc_scorer, which report average precision and ROC AUC scores, respectively.

We compared eight regression approaches and 11 classification approaches for this dataset. Some approaches used principal component analysis to reduce the number of features before training a model. Several approaches used meta-ensembles or stacking, and grid search was used to optimize hyperparameters for the meta-ensembles. Fig 2C and 2D summarizes the results of this analysis, and S1 Table includes histories for both the classification and regression analyses.

**References**

1. Ghandi M, Huang FW, Jané-Valbuena J, Kryukov GV, Lo CC, McDonald ER 3rd, et al. Next-generation characterization of the Cancer Cell Line Encyclopedia. Nature. 2019. doi:10.1038/s41586-019-1186-3
