# Supplementary material for: Galaxy-ML: An accessible, reproducible, and scalable machine learning toolkit for biomedicine
Source: PLoS Comput Biol. 2021 Jun 1;17(6):e1009014. doi: 10.1371/journal.pcbi.1009014 (PMC8213174; doi:10.1371/journal.pcbi.1009014)
Supplement: S4 Text — (DOCX) [file pcbi.1009014.s004.docx]

Using Galaxy-ML, we reproduced two deep learning models originally implemented in Selene, a deep learning library for biological sequence data [1]. The objective of the first analysis was to train an existing deep learning architecture using a novel dataset. Specifically, train the DeepSEA[2] architecture to model a tissue-specific regulatory element for a single transcription factor not supported by DeepSEA (see <https://github.com/FunctionLab/selene/tree/master/manuscript/case1>). To reproduce this analysis, we used the DeepSEA architecture (<https://github.com/FunctionLab/selene/blob/master/models/deepsea.py>), which contains 3 convolutional layers, 2 pooling layers, one fully connected layer and a Sigmoid output layer, plus the dataset used by Selene. Both Galaxy-ML and Selene obtained the same results: a ROC AUC of 0.94 (S2 Fig and S2 Table). The Galaxy history for this analysis used GPUs for training and evaluation, and it is available at <https://usegalaxy.eu/u/khanteymoori/h/sequencedlselenecase1v080522>

Another experiment run using Selene compared the performance of the DeepSEA architecture with an extended architecture that includes three additional convolutional layers (see <https://github.com/FunctionLab/selene/blob/master/manuscript/case2/README.md>). We reimplemented the extended architecture and then trained and evaluated this model using the Selene dataset, which included data for 919 regulatory elements. Our results are nearly identical to those obtained by Selene (S3 Fig and S2 Table). The Galaxy histories for this use-case are available from the following links:

- <https://figshare.com/articles/Galaxy-History-selene-case2_tar_gz/12398699> — fully trained over 100 epochs using a private Galaxy server with access to a GPU cluster;
- <https://usegalaxy.eu/u/qiang_gu/h/selene2> — online Galaxy history trained for only two epochs using CPU cluster.

**References**

1. Chen KM, Cofer EM, Zhou J, Troyanskaya OG. Selene: a PyTorch-based deep learning library for sequence data. Nat Methods. 2019;16: 315–318.

2. Zhou J, Troyanskaya OG. Predicting effects of noncoding variants with deep learning-based sequence model. Nat Methods. 2015;12: 931–934.
