# Supplementary material for: Galaxy-ML: An accessible, reproducible, and scalable machine learning toolkit for biomedicine
Source: PLoS Comput Biol. 2021 Jun 1;17(6):e1009014. doi: 10.1371/journal.pcbi.1009014 (PMC8213174; doi:10.1371/journal.pcbi.1009014)
Supplement: S1 Table — Each history/workflow ensures that an analysis can be completely reproduced because it lists all analysis steps and parameters. Each algorithm runs with two parameter configurations: default and best. Default configuration is a default value of parameters in Galaxy toolbox and best parameters are obtained by hyperparameter optimization. (DOCX) [file pcbi.1009014.s005.docx]

| Histories and Workflows | Algorithm | Parameters | | Links |
| --- | --- | --- | --- | --- |
|  |  | Default | Best |  |
| Classification | Penn | X |  | <https://usegalaxy.eu/u/qiang_gu/h/penn-classification-default> |
|  |  |  | X | <https://usegalaxy.eu/u/qiang_gu/h/penn-classification-best> |
|  | XGBoost | X |  | <https://usegalaxy.eu/u/kumara/w/xgbclassifierdefaultparams> |
|  |  |  | X | <https://usegalaxy.eu/u/kumara/w/xgbclassifierbestparams> |
| Regression | Adaboost | X |  | <https://usegalaxy.eu/u/kumara/w/adaboostregressor1905notuning> |
|  |  |  | X | <https://usegalaxy.eu/u/kumara/w/adaboostregressor1905> |
|  | Bagging | X |  | <https://usegalaxy.eu/u/kumara/w/baggingregressor1905notuning> |
|  |  |  | X | <https://usegalaxy.eu/u/kumara/w/baggingregressor1905> |
|  | BayesianRidge | X |  | <https://usegalaxy.eu/u/kumara/w/bayesianridgeregressor1905notuning> |
|  |  |  | X | <https://usegalaxy.eu/u/kumara/w/bayesianridgeregressor1905> |
|  | Decision Tree | X |  | <https://usegalaxy.eu/u/kumara/w/decisiontreeregressor1905notuning> |
|  |  |  | X | <https://usegalaxy.eu/u/kumara/w/decisiontreeregressor1905> |
|  | ElasticNet | X |  | <https://usegalaxy.eu/u/kumara/w/elasticnetregressor1905notuning> |
|  |  |  | X | <https://usegalaxy.eu/u/kumara/w/elasticnetregressor1905> |
|  | Extra Tree | X |  | <https://usegalaxy.eu/u/kumara/w/extratreeregressor1905notuning> |
|  |  |  | X | <https://usegalaxy.eu/u/kumara/w/extratreeregressor1905> |
|  | Extra Trees | X |  | <https://usegalaxy.eu/u/kumara/w/extratreesregressor1905notuning> |
|  |  |  | X | <https://usegalaxy.eu/u/kumara/w/extratreesregressor1905> |
|  | Gradient Boosting | X |  | <https://usegalaxy.eu/u/kumara/w/gradientboostingregressor1905notuning> |
|  |  |  | X | <https://usegalaxy.eu/u/kumara/w/gradientboostingregressor1905> |
|  | Huber | X |  | <https://usegalaxy.eu/u/kumara/w/huberregressor1905notuning> |
|  |  |  | X | <https://usegalaxy.eu/u/kumara/w/huberregressor1905> |
|  | KNN | X |  | <https://usegalaxy.eu/u/kumara/w/knnregressor1905notuning> |
|  |  |  | X | <https://usegalaxy.eu/u/kumara/w/knnregressor1905> |
|  | Linear | X |  | <https://usegalaxy.eu/u/kumara/w/linearregressor1905notuning> |
|  |  |  | X | <https://usegalaxy.eu/u/kumara/w/linearregressor1905> |
|  | Random Forest | X |  | <https://usegalaxy.eu/u/kumara/w/randomforestregressor1905notuning> |
|  |  |  | X | <https://usegalaxy.eu/u/kumara/w/randomforestregressor1905> |
|  | SVR | X |  | <https://usegalaxy.eu/u/kumara/w/linearsvrregressor1905notuning> |
|  |  |  | X | <https://usegalaxy.eu/u/kumara/w/supportvectorregressor1905> |
|  | XGBoost | X |  | <https://usegalaxy.eu/u/kumara/w/xgbregressor1905notuning> |
|  |  |  | X | <https://usegalaxy.eu/u/kumara/w/xgbregressor1905> |
