# Supplementary material for: Galaxy-ML: An accessible, reproducible, and scalable machine learning toolkit for biomedicine
Source: PLoS Comput Biol. 2021 Jun 1;17(6):e1009014. doi: 10.1371/journal.pcbi.1009014 (PMC8213174; doi:10.1371/journal.pcbi.1009014)
Supplement: S2 Table — All datasets used were obtained from Selene. AUPRC is the area under the precision-recall curve, and is also known as the average precision. “N.R.” means that the models did not report this information. (DOCX) [file pcbi.1009014.s006.docx]

|  | **Galaxy-ML** | | | **Selene** | |
| --- | --- | --- | --- | --- | --- |
| **Analysis/ Case Study** | **Balanced Accuracy** | **ROC AUC** | **AUPRC** | **ROC AUC** | **AUPRC** |
| 1 | 0.68 | 0.94 | 0.62 | 0.94 | N.R. |
| 2 | 0.62 | 0.928 | 0.365 | 0.938 | 0.362 |

**Table S2**. Performance results obtained using Galaxy-ML models fully trained using GPU and Selene models. All datasets used were obtained from Selene. AUPRC is the area under the precision-recall curve, and is also known as the average precision. “N.R.” means that the models did not report this information.
