# Supplementary figures and images for: Galaxy-ML: An accessible, reproducible, and scalable machine learning toolkit for biomedicine
Source: PLoS Comput Biol. 2021 Jun 1;17(6):e1009014. doi: 10.1371/journal.pcbi.1009014 (PMC8213174; doi:10.1371/journal.pcbi.1009014)

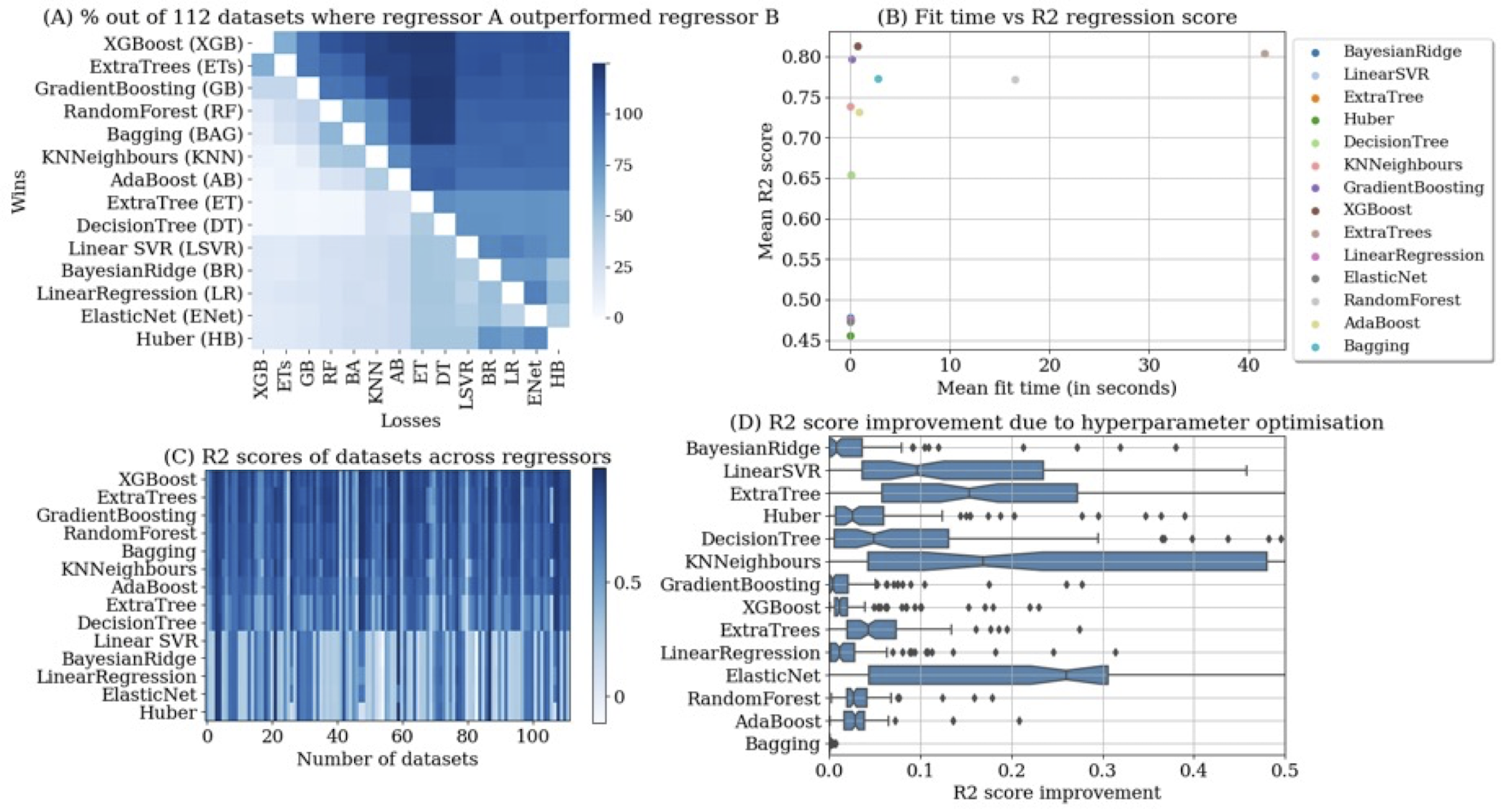

Supplement: S1 Fig — In panels A and C, heatmaps show the percentage of datasets for which the model listed along the row outperforms the model along the column. Panel A shows a heatmap in which each square contains a number of datasets for which the regressor on the left (wins) performed better than the regressor on the bottom (losses). For example, by mapping the color of the square between adaboost (shown on y-axis) and linear regression (LR) shown on x-axis to the adjacent color-scale, we conclude that the adaboost regressor performs better on 75–80 datasets (out of 112) than the linear regressor. The subplot also shows a comparison of different regressors (on y-axis). The ensemble regressors perform better on average than the other categories which include linear, tree and nearest neighbors regressors. Panel B shows a comparison between the running time and accuracy of different regressors. We compute an average running time of each regressor over all 112 datasets. The running time of a regressor on a dataset is the sum of the training and validation times for the best regression model. The regressors such as xgboost, gradient boosting and extra trees achieve > 0.80 r-squared score, but extra trees regressor requires significantly more time to finish compared to the other two regressors. Regressors such as linear regression, huber and elastic net are fast, but their accuracy is low. Decision and extra tree regressors are also fast, but their accuracy is better (> 0.7 r-squared score) than the linear regressors. Panel C shows the r-squared scores of each regressor for all datasets. The linear regressors at the bottom-left of the subplot achieve lower scores than the ensemble regressors such as xgboost, gradient boosting at top-left of the subplot. We can also see that for a few datasets, none of the regressors perform well. Panel D shows the importance of tuning the hyperparameters of the regressors for each dataset. It is not recommended to compute the performance of predictive alg [file pcbi.1009014.s007.tiff]

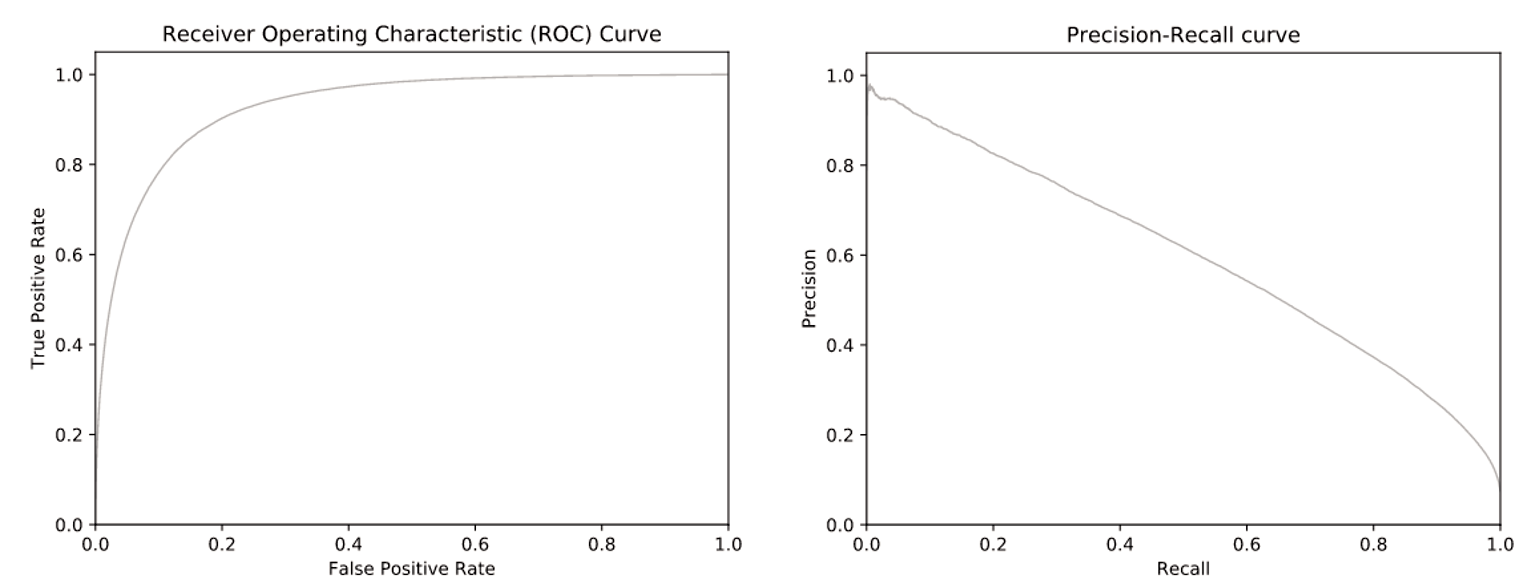

Supplement: S2 Fig — (TIFF) [file pcbi.1009014.s008.tiff]

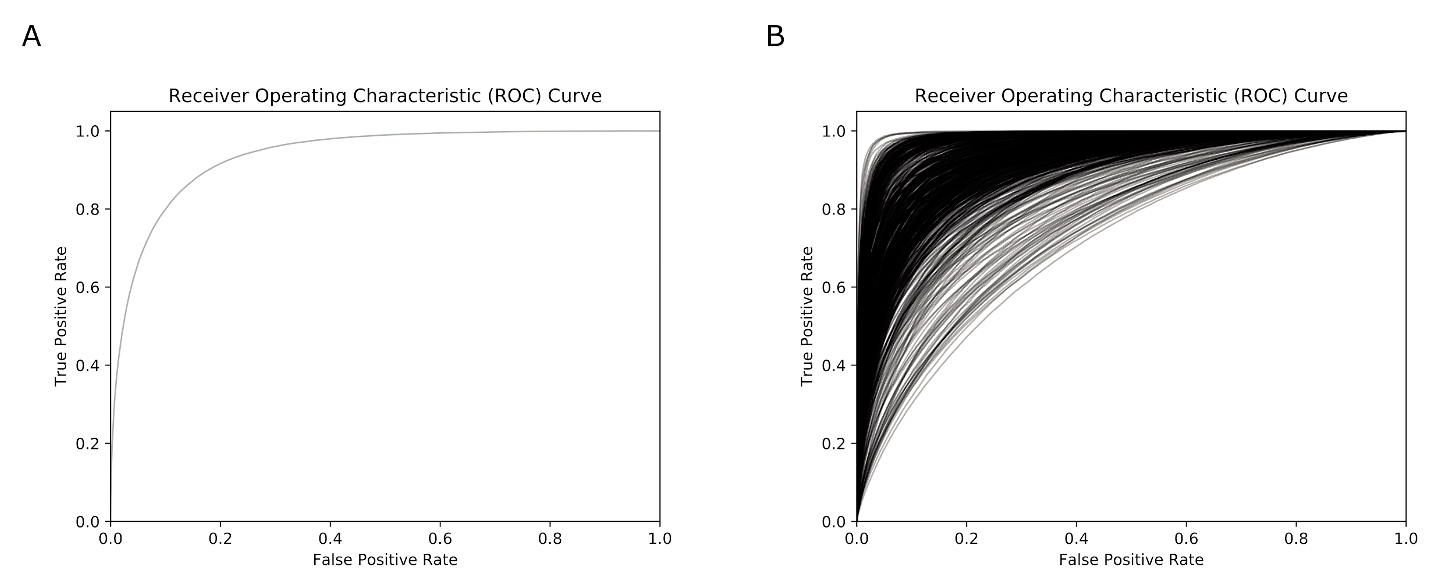

Supplement: S3 Fig — Visualized ROC curve results obtained from using the extended DeepSEA architecture [30] to model 919 regulatory elements: (A) average ROC for all elements and (B) individual ROC curves for each element. (TIFF) [file pcbi.1009014.s009.tiff]
