# Supplementary material for: Galaxy-ML: An accessible, reproducible, and scalable machine learning toolkit for biomedicine
Source: PLoS Comput Biol. 2021 Jun 1;17(6):e1009014. doi: 10.1371/journal.pcbi.1009014 (PMC8213174; doi:10.1371/journal.pcbi.1009014)
Supplement: Attachment — Submitted filename: Response to reviewers.docx [file pcbi.1009014.s010.docx]

**Response Overview:**

We thank the reviewers for their thoughtful critiques of our manuscript. We have revised the manuscript based on the concerns raised by the reviewers and, as a result, the revised manuscript is much stronger. Below we provide a point-by-point response to reviewer concerns. The revised manuscript includes highlighted and tracked changes to show where modifications were made.

**Reviewer #1 concerns**:

*The Galaxy platform itself has some limitations in terms of reproducibility. Although, the platform is openly available and hence the ML toolkit, it would be nice to see a more interoperable way of representing Galaxy workflows so they are repeatable in other cloud or local compute environments.*

*Extensibility is not necessarily clear or demonstrated in the publication. An potential minor addition, would be to address how additional ML libraries can be added to the existing toolkit and whether customization of those libraries is possible.*

Thank you for these suggestions. Addressing reproducibility inside and outside Galaxy and extensibility of Galaxy-ML is important given our goals of open and accessible computational science. We have added a new subsection in the Results section that discusses reproducibility and extensibility of Galaxy-ML. The most significant points in this subsection are:

1. There are ongoing efforts to enable reproducibility of Galaxy workflows outside of the Galaxy platform via interoperable workflows. The primary focus of these efforts are to make Galaxy compatible with workflows written in Common Workflow Language (CWL). CWL is a community standard for workflow definitions that is embraced by many workflow engines. When Galaxy’s CWL features are complete, Galaxy will be able to execute CWL workflows as well as export Galaxy workflows in CWL format. Once in CWL format, Galaxy workflows can be executed in any workflow engine that supports CWL, and this interoperability will extend to workflows composed of Galaxy-ML tools.
2. Extending Galaxy-ML with additional machine learning software libraries and custom methods is possible. Galaxy-ML has tools for data preprocessing, data generators, model definition, model training, and model evaluation, providing clear integration points where additional machine learning approaches can be added based on their functionality. These integration points mirror the scikit-learn application programming interface, which is widely used across the machine learning community. For instance, if a new library for creating gradient boosted decision trees becomes available, tools such as Model Fit and Hyperparameter Search can be augmented so users can create and use models from this library. As part of the use cases in the previous sections, we implemented custom modules for preprocessing, modeling, data splitting, and evaluation.

**Reviewer #2 concerns**:

*The statement “Galaxy-ML tools have also been run more than 12,000 times on the public U.S. (https://usegalaxy.org) and European Union Galaxy servers (https://usegalaxy.eu)” describes this toolkit as being run on both the US and EU servers, but I was unable to locate Galaxy-ML on the US servers with a moderate effort. The machine learning extensions for the EU server are located at https://ml.usegalaxy.eu, which I think is a useful URL the authors should consider adding to the manuscript, perhaps in lieu of the references to the primary server interfaces in this statement (which reference a broader set of tools for the EU server, and do not reference the Machine Learning tools at all on the US server). I would recommend the authors work with the US server team to incorporate the Galaxy-ML tools on that site as well, or provide some discussion in the manuscript on why they are not available on the US server. If the Galaxy-ML tools are not publicly available on the US server, the above statement should also be adjusted to reflect the reality of the situation (it is not useful to know that the authors somehow ran these tools on the US server if that is not available to the readers of this article).*

Thank you for these observations and suggestions. We’ve made several changes as a result of this feedback:

1. While the EU server has a “Machine Learning” tool category, the US server did not. We have now created a “Machine Learning” tool category on the U.S. server that can be found via browsing or search on the main Galaxy server’s tool panel. Unfortunately, the U.S. server does not yet support custom portals like the EU server.
2. We have added short instructions next to the server links in the statement quoted above with: (1) instructions on how to find the machine learning toolkit on the US server and (2) added the <https://ml.usegalaxy.eu> link for the EU server and added explanations about both links.

*The statement “To the best of our knowledge, this is among the most comprehensive machine learning experiments on cancer cell line RNA-seq and drug response datasets” is underqualified (to the best of our knowledge? among the most? how is comprehensiveness measured?), unnecessary (this does not add value to the paper or the useful work described there), and difficult to prove (large dataset ML experiments are a common research activity, particularly in cancer cell line data; many remain unpublished). This statement should be removed.*

We agree this sentence does not add to the manuscript and have removed it.
